# A Comparison of Pathogenicity and Virulence of Three Porcine Circovirus Type 2 (PCV2) Genotypes (a, b, and d) in Pigs Singularly Inoculated with PCV2 and Dually Inoculated with *Mycoplasma hyopneumoniae* and PCV2

**DOI:** 10.3390/pathogens10080979

**Published:** 2021-08-03

**Authors:** Taehwan Oh, Jeongmin Suh, Kee Hwan Park, Siyeon Yang, Hyejean Cho, Chanhee Chae

**Affiliations:** Department of Veterinary Pathology, College of Veterinary Medicine, Seoul National University, Gwanak-ro 1, Gwanak-gu, Seoul 08826, Korea; ohth93@gmail.com (T.O.); tobin1210@snu.ac.kr (J.S.); kylinhp@snu.ac.kr (K.H.P.); didtldus12@gmail.com (S.Y.); hcho21@snu.ac.kr (H.C.)

**Keywords:** *Mycoplasma hyopneumoniae*, porcine circovirus-associated disease, porcine circovirus type 2, PCV2 genotypes

## Abstract

The objective of this study was to compare the virulence of three different porcine circovirus type 2 (PCV2) genotypes (PCV2a, PCV2b, and PCV2d) in pigs infected with either one of these three PCV2 genotypes versus pigs dually inoculated with *Mycoplasma hyopneumoniae* and PCV2. Pigs were inoculated intratracheally with *M. hyopneumoniae* at 4 weeks of age followed by another intranasal inoculation at 6 weeks of age with one of three PCV2 genotypes. Dual infection with two pathogens produced moderate and severe dyspnea, lethargy, and reduced weight gain in pigs regardless of the PCV2 genotype evaluated compared with pigs only inoculated with PCV2. The overall levels of PCV2d viremia and severity of lymphoid lesions, and PCV2-antigen within lymphoid lesions were significantly higher in pigs dually inoculated with *M. hyopneumoniae*/PCV2d when compared with all other dually inoculated groups. The level of PCV2 viremia and the production of PCV2-associated lymphoid lesions did not differ significantly among PCV2a, PCV2b, and PCV2d single-inoculated pig groups. The results of this study demonstrated that *M. hyopneumoniae* potentiated the replication of PCV2d more than it did with the other PCV2 genotypes as measured by lymphoid lesion severity.

## 1. Introduction

Porcine circovirus type 2 (PCV2) and *Mycoplasma hyopneumoniae* are two of the major porcine pathogens and are responsible for enormous economic losses to pig producers [1,2]. PCV2 is highly prevalent worldwide [3]. It is the primary causative agent of porcine circovirus-associated disease (PCVAD), which collectively represents the many clinical manifestations of PCV2 infections such as postweaning multisystemic wasting syndrome (PMWS), PCV2-associated pneumonia, reproductive disorders, and enteric diseases [4]. *M. hyopneumoniae* is the primary causative agent of enzootic pneumonia. Enzootic pneumonia, a chronic respiratory disease, is widespread and characterized by reduced average daily weight gain and reduced feed conversion efficiency, while it simultaneously increases the predisposed animals to secondary infections [5].

PCVAD is considered a multifactorial disease whose full expression of clinical disease is reached by a variety of infectious agents as cofactors [3]. PCV2 infection is a necessary one of these cofactors but is not always sufficient in causing PCVAD by itself. *M. hyopneumoniae* is frequently found as a co-infectious agent in PCVAD [2,6] and has been shown to enhance PCV2-associated disease when administered two weeks before PCV2 infection [7]. PCV2a has been the only genotype previously studied in the co-infection model with *M. hyopneumoniae* [7]. PCV2a, PCV2b, and PCV2d currently remain the most prevalent genotypes in worldwide swine herd circulation [8]. Therefore, the objective of this study was to compare the virulence and pathogenicity of PCVAD reproduced by pigs co-infected with each of the three PCV2 genotypes and *M. hyopneumoniae*.

## 2. Results

### 2.1. Clinical Signs

**Figure 1:**
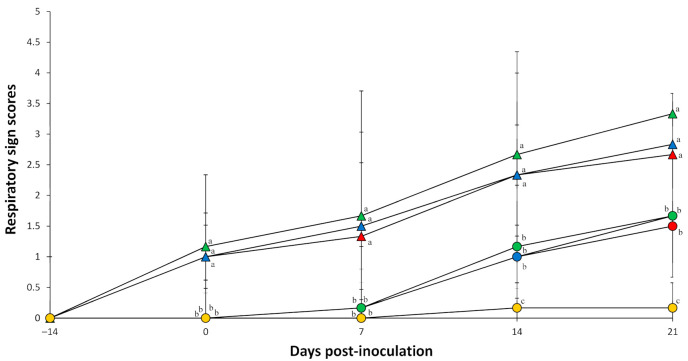
Respiratory sign scores of pigs dually inoculated with *Mycoplasma hyopneumoniae*/PCV2a (▲), *M. hyopneumoniae*/PCV2b (▲), and *M. hyopneumoniae*/PCV2d (▲), and pigs singularly inoculated with PCV2a (●), PCV2b (●), and PCV2d (●), and negative control pigs (●). Variation is expressed as the standard deviation. Different superscripts (a, b and c) indicate significant (*p* < 0.05) difference among seven groups.

All pigs inoculated with a single form of PCV2 exhibited signs of minimal respiratory disease and remained clinically normal. Meanwhile, clinical signs were observed in all pigs dually inoculated with either *M. hyopneumoniae*/PCV2a, *M. hyopneumoniae*/PCV2b, or *M. hyopneumoniae*/PCV2d. Regardless of the PCV2 genotype, pigs dually inoculated with *M. hyopneumoniae*/PCV2 developed severe respiratory distress by 7 days post-inoculation (dpi) that continued through 21 dpi. The respiratory sign scores at 7 to 21 dpi in pigs dually inoculated with *M. hyopneumoniae*/PCV2a, *M. hyopneumoniae*/PCV2b, and *M. hyopneumoniae*/PCV2d were significantly (*p* < 0.05) higher compared with those of pigs singularly inoculated with PCV2a, PCV2b, or PCV2d only and those of negative control pigs (Figure 1). All dually inoculated pigs developed a rough hair coat, regardless of the PCV2 genotype administered during infection. No clinical signs were observed in negative controls throughout the entire experiment.

### 2.2. Growth Performance

A statistical difference was not observed at the start of the experiment (28-day-old pigs) in terms of average body weight among the seven groups. Pigs dually inoculated with *M. hyopneumoniae*/PCV2a, *M. hyopneumoniae*/PCV2b, and *M. hyopneumoniae*/PCV2d had significantly (*p* < 0.05) lower average body weights compared to pigs inoculated with either PCV2a, PCV2b or PCV2d alone, as well as negative controls at 63 days old (21 dpi). Pigs dually inoculated with *M. hyopneumoniae*/PCV2a, *M. hyopneumoniae*/PCV2b, and *M. hyopneumoniae*/PCV2d had a significantly lower (*p* < 0.05) average daily weight gain (ADWG; gram/pig/day) between 28 and 63 days of age compared to pigs singularly inoculated with either PCV2a, PCV2b, PCV2d, or the negative controls (Table 1).

### 2.3. Enzyme-Linked Immunosorbent Assay

**Figure 2:**
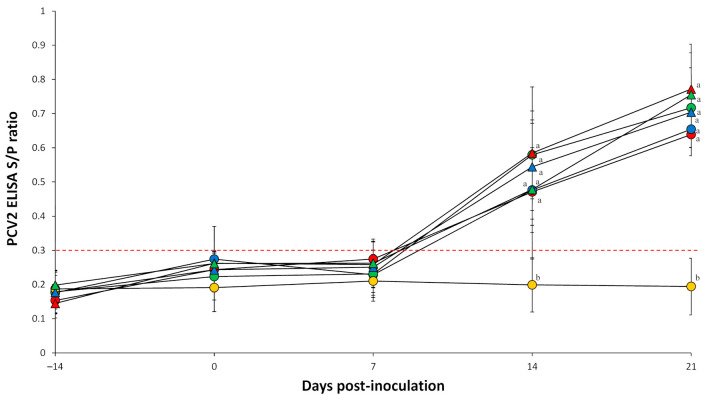
Porcine circovirus type 2 (PCV2)-specific ELISA antibody levels in serum of pigs dually inoculated with *Mycoplasma hyopneumoniae*/PCV2a (▲), *M. hyopneumoniae*/PCV2b (▲), and *M. hyopneumoniae*/PCV2d (▲), and pigs singularly inoculated with PCV2a (●), PCV2b (●), and PCV2d (●), and negative control pigs (●). Variation is expressed as the standard deviation. Serum samples are considered positive for PCV2 antibodies if the optical density (OD) is greater than 0.3 (red dotted line) Different superscripts (a and b) indicate significant (*p* < 0.05) difference among 7 groups.

Pigs dually inoculated with *M. hyopneumoniae*/PCV2a, *M. hyopneumoniae*/PCV2b, and *M. hyopneumoniae*/PCV2d and pigs singularly inoculated with PCV2a, PCV2b, and PCV2d had a significantly (*p* < 0.05) higher S/P ratio at 14 and 21 dpi compared to negative control pigs (Figure 2). PCV2 antibodies were not detected in negative control pigs throughout the entire experiment.

### 2.4. Quantification of PCV2 DNA in Blood

**Figure 3:**
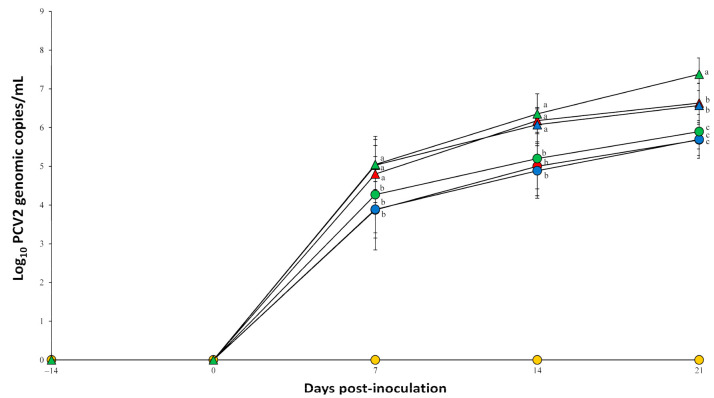
Mean values of the genomic copy number of porcine circovirus type 2 (PCV2) DNA in serum of pigs dually inoculated with *Mycoplasma hyopneumoniae*/PCV2a (▲), *M. hyopneumoniae*/PCV2b (▲), and *M. hyopneumoniae*/PCV2d (▲), and pigs singularly inoculated with PCV2a (●), PCV2b (●), and PCV2d (●), and negative control pigs (●). Variation is expressed as the standard deviation. Different superscripts (a, b, and c) indicate significant (*p* < 0.05) difference among seven groups.

Prior to challenge, all serum samples collected from pigs in all seven groups tested negative for PCV2a, PCV2b, and PCV2d. Pigs dually inoculated with *M. hyopneumoniae*/PCV2a, *M. hyopneumoniae*/PCV2b, and *M. hyopneumoniae*/PCV2d had a significantly (*p* < 0.05) higher number of PCV2 genomic copies compared to pigs singularly inoculated with either PCV2a, PCV2b or PCV2d at 7, 14, and 21 dpi. Pigs dually inoculated with *M. hyopneumoniae*/PCV2d had a significantly (*p* < 0.05) higher number of PCV2 genomic copies compared to pigs dually inoculated with *M. hyopneumoniae*/PCV2a, and *M. hyopneumoniae*/PCV2b at 21 dpi (Figure 3). PCV2 genomic copies were not detected in negative control pigs throughout the entire experiment.

### 2.5. Quantification of Mycoplasma hyopneumoniae DNA in Larynx

**Figure 4:**
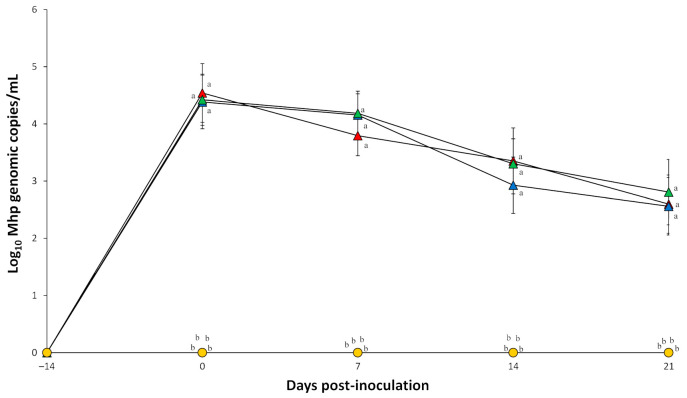
Mean values of the genomic copy number of *Mycoplasma hyopneumoniae* (Mhp) in larynx of pigs dually inoculated with *M*. *hyopneumoniae*/PCV2a (▲), *M. hyopneumoniae*/PCV2b (▲), and *M. hyopneumoniae*/PCV2d (▲), and pigs singularly inoculated with PCV2a (●), PCV2b (●), and PCV2d (●), and negative control pigs (●). Variation is expressed as the standard deviation. Different superscripts (a and b) indicate significant (*p* < 0.05) difference among 7 groups.

Prior to challenge, all serum samples collected from pigs tested negative for *M. hyopneumoniae*. *M. hyopneumoniae* genomic copies were detected in pigs dually inoculated with either *M. hyopneumoniae*/PCV2a, *M. hyopneumoniae*/PCV2b, or *M. hyopneumoniae*/PCV2d throughout the entire experiment. No significant differences in the number of *M. hyopneumoniae* genomic copies were detected in pigs dually inoculated with *M. hyopneumoniae*/PCV2a, *M. hyopneumoniae*/PCV2b, and *M. hyopneumoniae*/PCV2d (Figure 4). *M. hyopneumoniae* genomic copies were not detected in pigs singularly inoculated with either PCV2a, PCV2b, or PCV2d, and in negative control pigs throughout the entire experiment.

### 2.6. Histopathology

**Figure 5:**
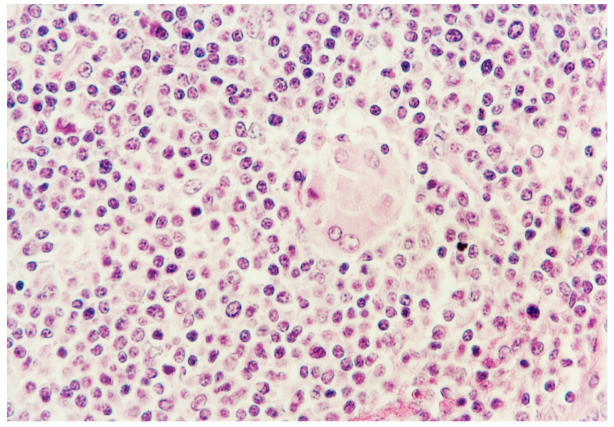
Granulomatous foci characterized by infiltration with reactive histiocytes and multinucleated giant cells from pigs dually inoculated with *Mycoplasma hyopneumoniae*/PCV2d.

Mild histopathologic lesions were seen in piglets singularly inoculated with either PCV2a, PCV2b, or PCV2d. Histopathologic lesions in pigs dually inoculated with *M. hyopneumoniae*/PCV2a, *M. hyopneumoniae*/PCV2b, and *M. hyopneumoniae*/PCV2d were severe and widespread in the lymph nodes. The primary lesion unanimously found was widespread disseminated granulomatous inflammation found mainly in lymph nodes and spleens, and occasionally in kidneys. A moderate to severe infiltrate of lymphocytes, neutrophils, and eosinophils accompanied the macrophages. Lymph nodes were depleted of mature lymphocytes, and germinal centers were either reduced or absent. Multinucleated giant cells were prominent in pigs dually inoculated with *M. hyopneumoniae*/PCV2a, *M. hyopneumoniae*/PCV2b, and *M. hyopneumoniae*/PCV2d (Figure 5). Pigs dually inoculated with *M. hyopneumoniae*/PCV2a, *M. hyopneumoniae*/PCV2b, and *M. hyopneumoniae*/PCV2d had moderate-to-severe peribronchiolar and perivascular lymphoid tissue hyperplasia, moderate-to-severe lymphohistiocytic inflammation in the lamina propria of airways, and mixed inflammation in the lumina of the airways. Tissue samples from negative control pigs were histologically normal.

Pigs dually inoculated with *M. hyopneumoniae*/PCV2a, *M. hyopneumoniae*/PCV2b, and *M. hyopneumoniae*/PCV2d had significantly higher (*p* < 0.05) microscopic lymphoid lesion scores compared to pigs singularly inoculated with PCV2a, PCV2b, and PCV2d alone at 21 dpi. Pigs dually inoculated with *M. hyopneumoniae*/PCV2d had significantly higher (*p* < 0.05) microscopic lymphoid lesion scores compared to pigs dually inoculated with *M. hyopneumoniae*/PCV2a and *M. hyopneumoniae*/PCV2b at 21 dpi (Table 2).

### 2.7. Immunohistochemistry

Regardless of the PCV2 genotype, all pigs infected with PCV2, either alone or in combination with *M. hyopneumoniae* reacted positively for PCV2 antigen. Lymph nodes contained prominent accumulations of viral antigen were detected chiefly in follicular macrophages found in collapsed germinal centers. Pigs dually inoculated with *M. hyopneumoniae*/PCV2a, *M. hyopneumoniae*/PCV2b, and *M. hyopneumoniae*/PCV2d had a significantly (*p* < 0.05) higher number of PCV2-positive cells per unit of tissue in their lymph nodes than pigs singularly inoculated with PCV2a, PCV2b, or PCV2d. Pigs dually inoculated with *M. hyopneumoniae*/PCV2d had a significantly (*p* < 0.05) higher number of PCV2-positive cells per unit of tissue in their lymph nodes than pigs dually inoculated with *M. hyopneumoniae*/PCV2a and *M. hyopneumoniae*/PCV2b (Table 2). PCV2 antigens were not detected in any lymph nodes examined from negative control pigs.

### 2.8. Correlation between PCV2 Viremia and Lymphoid Lesions

Positive and significant correlations were found in pigs dually inoculated with PCV2 and *M. hyopneumoniae* groups (*M. hyopneumoniae*/PCV2a: R = 0.941, *p* < 0.05; *M. hyopneumoniae*/PCV2b: R = 0.883, *p* < 0.05; *M. hyopneumoniae*/PCV2d: R = 0.880, *p* < 0.05) between PCV2 viral blood load and the severity of lymphoid lesions.

## 3. Discussion

Unlike the pigs infected with a single genotype of PCV2, a dual infection with *M. hyopneumoniae*/PCV2 resulted in the reproduction of PCVAD across all three PCV2 genotypes. Pigs dually infected with *M. hyopneumoniae*/PCV2 met the criteria of PCVAD classification such as typical clinical signs including ADWG, pathological lesions and presence of PCV2 antigen within lymphoid lesions. The present results are consistent with previous studies, where pigs dually infected with PCV2a and *M. hyopneumoniae* induced the full clinical manifestation of PCVAD [7]. This study demonstrated that PCV2b and PCV2d are also able to develop PCVAD (along with the previously established PCV2a) when co-infection with *M. hyopneumoniae* occurs.

In the present study, *M. hyopneumoniae* clearly potentiates PCV2-associated disease in dually infected pigs. Nevertheless, the specific mechanisms by which *M. hyopneumoniae* enhances PCV2-associated lesions have not been determined. *M. hyopneumoniae* is traditionally considered a strict extracellular pathogen and the site of infection remains limited mainly at the lung and cilia of bronchial and bronchiolar lining epithelial cells [9,10]. It has, however, been cultured on occasion from the liver, spleen, kidneys, and bronchial lymph nodes of pigs experimentally infected with *M. hyopneumoniae* [11,12]. The inflammatory response, a hallmark of lung infection caused by *M. hyopneumoniae*, may be a pivotal mechanism in providing the PCV2 organism with the ability to invade and survive in alveolar macrophages for further dissemination to the liver, spleen, kidneys, and lymph node [11,12,13]. It is therefore possible that PCV2 and *M. hyopneumoniae* can interact with each other in the same or neighboring infected macrophage, promoting high levels of replication of PCV2 in lymphoid tissues.

Pigs dually infected with *M. hyopneumoniae*/PCV2d produced significantly higher levels of PCV2 loads in blood and lymph nodes with more severe lymphoid lesions at 21 dpi only when compared with pigs dually infected with either *M. hyopneumoniae*/PCV2a or *M. hyopneumoniae*/PCV2b. Nonetheless, there are no significant differences in serology, levels of PCV2 loads in blood at other points, and ADWG among PCV2 genotypes in pigs dually infected with *M. hyopneumoniae* and PCV2. It currently cannot be explained with complete certainty as to why *M. hyopneumoniae* potentiates the replication of PCV2d more than other PCV2 genotypes, leading to a greater severe lymphoid lesion severity. This dual infection is based on the sequential model, which is *M. hyopneumoniae* infection followed 14 days later by PCV2 infection [7]. In *M. hyopneumoniae*-infected pigs, cells positive for interferon-γ (IFN-γ) were detected at 7 dpi, with their numbers increasing at 14 and 21 dpi, and slightly decreasing thereafter [14]. IFN-γ is known as pro-inflammatory cytokine and enhances the replication of PCV2 [15]. PCV2d has one additional amino acid (lysine) in its capsid protein encoded by ORF2 compared to PCV2a and PCV2b. In particular, the capsid protein of PCV2 is involved in enhancing viral replication in vitro [16]. Moreover, pigs experimentally infected with PCV2d resulted in higher levels of PCV2 viremia and a greater lymphoid lesion severity compared with pigs experimentally infected with either PCV2a or PCV2b [17]. It is speculated that this genetic difference between PCV2d and PCV2a/PCV2b may enhance PCV2d replication in the pathological conditions induced by *M. hyopneumoniae* infection. Additional studies are necessary to elucidate the replication preference of PCV2d by *M. hyopneumoniae*.

This experiment is to compare the virulence among the three PCV2 genotypes. Therefore, *M. hyopneumoniae* used merely as co-factor to induce full manifestation of PCVAD. In addition, there were no significant differences in the levels of *M. hyopneumoniae* laryngeal loads in the pigs dually infected with *M. hyopneumoniae*/PCV2a, *M. hyopneumoniae*/PCV2b, and *M. hyopneumoniae*/PCV2d. Since the effect of PCV2 on *M. hyopneumoniae* was not determined in this study, it is not necessary to include the pigs singularly infected with *M. hyopneumoniae*, in order to avoid unnecessary usage of pigs. Further studies are needed to determine the effect of PCV2 on *M. hyopneumoniae* including *M. hyopneumoniae*-infected group.

This is the first pathogenicity and virulence comparative study of PCV2a, PCV2b and PCV2d genotypes experimentally infected into pigs both independently and as a co-infection with *M. hyopneumoniae*. The results of this study are clinically relevant as PCV2d is the predominant genotype in Asian and North American swine populations [13,18,19,20,21,22]. A Chinese PCV2d strain can result in more severe disease compared to PCV2a or PCV2b infections [17]. In contrast, Korean and North American isolates for three PCV2 genotypes (a, b, and d) reported no significant difference in virulence [23,24]. This study has been determined the virulence with only one strain for each PCV2 genotype and hence differences in virulence may be due solely to strain rather than genotypes. Therefore, caution should be taken in interpreting comparative virulence among the three PCV2 genotypes. Further studies are needed to compare the virulence using different strains of PCV2a, PCV2b, and PCV2d genotypes.

## 4. Materials and Methods

### 4.1. Animals

Forty-two clinically healthy, colostrum-fed conventional pigs from sows that had not been previously vaccinated against PCV2 were purchased at 25 days of age from a commercial farm that was free of PRRSV. The farm was also *M. hyopneumoniae*-free based on serological testing, long-term clinical and slaughter history. The farm was seropositive for PCV2 but did not show PCVAD. Pigs were seronegative for PRRSV (HerdChek PRRS X3 Ab test, IDEXX Laboratories Inc., Westbrook, ME, USA), PCV2 (INgezim CIRCO IgG, Ingenasa, Madrid, Spain), and *M. hyopneumoniae* (*M*. *hyo*. Ab test, IDEXX Laboratories Inc.). Piglets were confirmed negative for PCV2 (a, b, and d) and PRRSV viremia, and *M. hyopneumoniae* laryngeal shedding by real-time polymerase chain reaction (PCR) testing upon arrival [25,26,27,28].

### 4.2. Experimental Design

For the study, pigs were allocated into 7 groups (6 pigs per group) using the random number generator function from Excel (Microsoft Corporation, Redmond, WA, USA) (Table 1). A minimum sample size per each group was calculated using pwr package in R version 4.1.0 (R Core Team: a language and environment for statistical computing. R Foundation for Statistical Computing, Vienna, Austria, http://www.r.project.org, accessed on 25 July 2021). A 0.05 significance level, 0.4 effect size, and 70% power were used to calculate the minimum number of piglets needed per group. This value was determined as 5.67, therefore, at least six pigs were designated per group.

Pigs in each group were randomly assigned into seven separate rooms. At −14 dpi (28 days of age), pigs in the three *M. hyopneumoniae*/PCV2 groups were intramuscularly anesthetized with a mixture of 2.2 mg/kg xylazine hydrochloride (Rompun, Bayer Healthcare, Shawnee Mission, KS, USA), 2.2 mg/kg tiletamine hydrochloride, and 2.2 mg/kg zolazepam hydrochloride (Zoletil 50, Virbac, Carros, France). Pigs were then inoculated intratracheally with 7 mL of *M. hyopneumoniae* (strain SNU98703) culture medium containing 10^7^ color changing units (CCU)/mL. Experimental inoculation of pigs with *M. hyopneumoniae* (strain SNU98703) induced mycoplasmal pneumonia in the lungs [29].

At 0 dpi (42 days of age), pigs in the three *M. hyopneumoniae*/PCV2 groups were each inoculated intranasally with either 3 mL of PCV2a (SNUVR100032 strain, 5th passage in PCV-free PK15 cell lines, PCV-free PK cell line was kindly provided by WOOGENE B&G Ltd., Seoul, Korea), PCV2b (SNUVR202155, 5th passage in PCV-free PK15 cell lines), or PCV2d (SNUVR202003, 5th passage in PCV-free PK15 cell lines) inoculum containing 1.2 × 10^5^ 50% tissue culture infective dose (TCID_50_/mL), based on their groups. Pigs in the PCV2a, PCV2b, and PCV2d groups were each inoculated intranasally with 3 mL of PCV2a, PCV2b, and PCV2d, respectively, as mentioned above. Pigs in the negative control group were inoculated intranasally with 3 mL of phosphate-buffered saline (PBS, 0.01M, pH 7.4).

Blood samples were collected from each pig by jugular venipuncture at −14, 0, 7, 14, and 21 dpi. Pigs were sedated by an intravenous injection of sodium pentobarbital and then euthanized by electrocution at 21 dpi as previously described [30]. Tissues were collected from each pig at necropsy.

### 4.3. Clinical Observation

Pigs were monitored daily for clinical signs and scored weekly using a score ranking system that ranged from 0 (normal) to 6 (severe dyspnea and abdominal breathing) [31]. All observers involved in these processes were blinded to the type of challenge virus.

### 4.4. Growth Performance

The live weight of each pig was measured at 28 (−14 dpi) and 63 (21 dpi) days of age. ADWG was analyzed over the time period between 28 and 63 days of age. ADWG during the different production stages was calculated as the difference between the starting and final weight divided by the duration of the stage.

### 4.5. Quantification of PCV2 DNA

Serum samples from all experimental groups were collected at −14, 0, 7, 14, and 21 dpi. A commercial kit (QIAamp DNA Mini Kit, QIAGEN, Valencia, CA, USA) was used to extract DNA from serum samples for PCV2. Genomic DNA copy numbers for PCV2a, PCV2b, and PCV2d were quantified by real-time PCR [25,26].

### 4.6. Quantification of M. hyopneumoniae DNA

Laryngeal swab samples from all experimental groups were collected at −14, 0, 7, 14, and 21 dpi. A commercial kit (QIAamp DNA Mini Kit, QIAGEN) was used to extract DNA from laryngeal swabs for *M. hyopneumoniae*. The number of genomic DNA copies for *M. hyopneumoniae* was then quantified by real-time PCR [27].

### 4.7. Serology

Serum samples were also tested for antibodies against PCV2 (INgezim CIRCO IgG, Ingenasa). Serum samples were considered positive for PCV2 antibodies if the optical density (OD) was greater than 0.3 according to the manufacturer’s instructions.

### 4.8. Histopathology

For the morphometric analysis of histopathological changes in superficial inguinal lymph nodes, three sections of that lymph node were examined “blindly” [32]. Lymph nodes were evaluated for presence of lymphoid depletion and inflammation, and given a score ranging from 0 to 5 (0 = normal; 1 = mild lymphoid depletion; 2 = mild to moderate lymphoid depletion and histiocytic replacement; 3 = moderate diffuse lymphoid depletion and histiocytic replacement; 4 = moderate to severe lymphoid depletion and histiocytic replacement; 5 = severe lymphoid depletion and histiocytic replacement).

### 4.9. Immunohistochemistry

Immunohistochemistry (IHC) and morphometric analysis of IHC were carried out as previously described [33]. Positive signal was quantified using the NIH Image J 1.45s Program (http://imagej.nih.gov/ij/download.html, accessed on 25 July 2021). For each slide of lymph node tissue, 10 fields were randomly selected, and the number of positive cells per unit area (0.25 mm^2^) was counted. The mean values were also calculated [33].

### 4.10. Statistical Analysis

Prior to statistical analysis, real-time PCR data were log-transformed to reduce variance and positive skewness. Data were tested for normal distribution using the Shapiro-Wilk test. A one-way analysis of variance (ANOVA) was used to examine whether significant statistical differences existed among the seven groups for each time point. When a one-way ANOVA test result showed statistical significance, a post-hoc test was conducted for a pairwise comparison with Tukey’s adjustment. If the normality assumption was not met, the Kruskal-Wallis test was performed. When the result from the Kruskal Wallis test showed statistical significance, a Mann-Whitney test with Tukey’s adjustment was performed to compare the differences among the groups. Spearman’s (non-normally distributed variables) correlations were applied to determine the relationship between the levels of PCV2 viremia and severity of lymphoid lesions. A value of *p* < 0.05 was considered to be significant.

## Figures and Tables

**Table 1:** Body weight and average daily weight gain (ADWG) data (mean ± standard deviation) from pigs dually infected with *Mycoplasma hyopneumoniae* and porcine circovirus type 2 (PCV2), and pigs singularly infected with PCV2 at 28 days of age (−14 days post-inoculation, dpi) and 63 days of age (21 dpi).

	Body Weight (kg)		ADWG (Gram/Pig/Day)
Groups	28 Days Old (−14 dpi)	63 Days Old (21 dpi)	between 28 and 63 Days Old
*M. hyopneumoniae*/PCV2a	6.47 ± 0.40	14.92 ± 0.61 ^a^	241.43 ± 21.44 ^a^
*M. hyopneumoniae*/PCV2b	6.50 ± 0.24	15.02 ± 0.63 ^a^	243.33 ± 25.39 ^a^
*M. hyopneumoniae*/PCV2d	6.42 ± 0.35	14.80 ± 1.07 ^a^	239.52 ± 36.96 ^a^
PCV2a	6.47 ± 0.34	17.00 ± 0.93 ^b^	300.95 ± 27.62 ^b^
PCV2b	6.28 ± 0.38	16.97 ± 0.97 ^b^	305.24 ± 25.26 ^b^
PCV2d	6.38 ± 0.39	16.93 ± 1.01 ^b^	301.43 ± 21.51 ^b^
Negative control	6.53 ± 0.32	18.02 ± 0.75 ^b^	328.10 ± 18.73 ^b^

Different superscripts (a and b) indicate significant (*p* < 0.05) difference among 7 groups.

**Table 2:** Pathology data (mean ± standard deviation) from pigs dually inoculated with *Mycoplasma hyopneumoniae* and porcine circovirus type 2 (PCV2), and pigs singularly inoculated with PCV2 at 21 days post-inoculation.

Groups	Microscopic Lymphoid Lesion Scores (Ranged from 0 to 5)	PCV2-Antigen Positive Cells Within Lymphoid Lesion
*M. hyopneumoniae*/PCV2a	3.10 ± 0.28 ^a^	34.61 ± 6.78 ^a^
*M. hyopneumoniae*/PCV2b	3.13 ± 0.45 ^a^	34.83 ± 6.46 ^a^
*M. hyopneumoniae*/PCV2d	3.87 ± 0.50 ^b^	45.39 ± 4.88 ^b^
PCV2a	1.80 ± 0.25 ^c^	22.17 ± 4.57 ^c^
PCV2b	1.73 ± 0.50 ^c^	21.28 ± 6.33 ^c^
PCV2d	1.77 ± 0.34 ^c^	22.72 ± 7.17 ^c^
Negative control	0 ± 0	0 ± 0

Different superscripts (a, b, and c) indicate significant (*p* < 0.05) difference among 7 groups.

## Data Availability

The data present in the study are available on request from the corresponding authors.
